# Controlled Growth of Silver Oxide Nanoparticles on the Surface of Citrate Anion Intercalated Layered Double Hydroxide

**DOI:** 10.3390/nano11020455

**Published:** 2021-02-11

**Authors:** Do-Gak Jeung, Minseop Lee, Seung-Min Paek, Jae-Min Oh

**Affiliations:** 1 Department of Energy and Materials Engineering, Dongguk University-Seoul, Seoul 04620, Korea; jdk941101@gmail.com; 2 Department of Chemistry, Kyungpook National University, Daegu 41566, Korea; shlee6697@naver.com

**Keywords:** silver oxide, nanoparticle, layered double hydroxide, crystal growth, antibacterial

## Abstract

Silver oxide nanoparticles with controlled particle size were successfully obtained utilizing citrate-intercalated layered double hydroxide (LDH) as a substrate and Ag^+^ as a precursor. The lattice of LDH was partially dissolved during the reaction by Ag^+^. The released hydroxyl and citrate acted as a reactant in crystal growth and a size controlling capping agent, respectively. X-ray diffraction, X-ray photoelectron spectroscopy, and microscopic measurements clearly showed the development of nano-sized silver oxide particles on the LDH surface. The particle size, homogeneity and purity of silver oxide were influenced by the stoichiometric ratio of Ag/Al. At the lowest silver ratio, the particle size was the smallest, while the chemical purity was the highest. X-ray photoelectron spectroscopy and UV-vis spectroscopy results suggested that the high Ag/Al ratio tended to produce silver oxide with a complex silver environment. The small particle size and homogeneous distribution of silver oxide showed advantages in antibacterial efficacy compared with bulk silver oxide. LDH with an appropriate ratio could be utilized as a substrate to grow silver oxide nanoparticles with controlled size with effective antibacterial performance.

## 1. Introduction

Silver oxide nanoparticles (SONPs) have been widely studied due to their unique optoelectronic property and high antibacterial activities. As a semiconducting material, SONPs are known to have a wide band-gap from 1.3 to 2.4 eV [1,2,3], making them suitable for photocatalytic applications, especially with the visible light-harvesting ability [4,5,6,7,8]. The SONP itself is utilized in photoelectrochemical or photocatalytic applications. Furthermore, SONP-based ternary oxides show fine-tuned catalytic performance as the variable coordination environments made by multi-components influence electronic structure and redox behaviour [9]. In general, SONP is synthesized through a precipitation method in which Ag^+^(aq) is titrated with an alkaline solution, e.g., NaOH(aq), to obtain silver oxide (Ag_2_O) [10,11,12]. Although precipitation is the simplest method to produce Ag_2_O, ion sources can remain unreacted, resulting in a non-stoichiometric problem. As an alternative for the precipitation method, a sol–gel route can be utilized [13]. As the sol–gel process involves the conversion of monomers into sol that acts as the precursor for gel, all the reactants are integrated into the final product.

The synthesis of Ag_2_O is fairly simple as the reaction spontaneously occurs through acid (Ag^+^)-base (O^2−^) adduct formation. However, the fabrication of SONPs with a targeted size and shape is another challenging topic. The particle size of SONPs determines the electronic structure and it affects the interaction toward complementary catalyst or substrate, finally influencing the overall catalytic performance [14,15]. The electronic structure of semiconductors, such as SONPs, has two band structures, the valence and conduction bands. They are separated by a band-gap of appropriate energy. As the size of semiconducting materials reduces, the number of atomic orbitals participating in the band structure decreases, and the energy difference between the two bands increases. In addition, the particle morphology of SONPs is reported to strongly affect the antibacterial property as the release of Ag^+^, the antibacterial agent, was dependent on the size and shape of SONP. It was suggested by Wang et al., that different shapes of SONP particles resulted in different Ag^+^ dissolution [16]. For example, the octahedron-shaped particles which contained Ag^+^ ions sandwiched by two planes of O_2_^−^ ions retarded Ag^+^ dissolution; however, the cubic-shaped particle which had alternate stacking of Ag^+^ ions and O_2_^−^ ions, resulted in the facilitated release of Ag^+^. Furthermore, the size effect is also an important factor in antibacterial ability. As the particle size decreases, the surface area to volume ratio increases. The large specific surface area accelerated the release of Ag^+^ ions and antibacterial effect [17]. The Ag^+^ ions suppress bacteria by various routes including generation of reactive oxygen species (ROS) and direct coordination with DNA base pairs [18].

Although the particle morphology of SONP is important, difficulties in SONP synthesis with controlled particle size and homogeneity have often been reported, due to the fast precipitation reaction speed [16,19]. The general chemical properties of the products can be controlled by experimental parameters including reagent concentration and stoichiometry. However, their detailed physical properties, such as particle size, surface morphology and uniformity of particles, are not easily controlled by simple chemical control [20,21]. Due to these limitations, there are several proposed complementary steps in addition to the precipitation process. For instance, SONP particles with controlled size could be prepared by microwave irradiation with adjustable temperature over a short period [22]. Reverse micelle methods that limit crystal growth by preventing particle agglomeration can be adopted [23]. The electrical discharge process, which controls the generation of highly dispersed silver cations, was known to produce uniformly distributed SONPs [24]. As it is possible to micro-manipulate reaction conditions during sputtering or anodization, these methods have also been proposed to prepare homogeneous SONPs with accurately controlled coverage and loading content. Oxidation of size-controlled Ag nanoparticles as precursors was suggested as an alternative strategy to produce SONPs with high homogeneity [25,26]. As an oxidation process involves direct conversion, the uniform size of the precursor was maintained in SONPs.

SONPs are often combined with other components to form hybrid catalysts or effective antibacterial surface coating [17,19,27]. For example, SONPs hybridized with ZnO showed promoted interfacial electron transfer process and reduced the charge recombination, resulting in improved photocatalytic effect [19]. Due to the antibacterial property and high stability, SONPs can be utilized as an infection-free coating in medical materials [17,27]. Inspired by the hybridization strategy, we propose a methodology to prepare SONPs with controlled size through hybridization with an appropriate substrate. A similar approach was reported by Rajabi et al. [17]. They applied an ultrasonic method to develop SONPs with homogeneous particle size on fabric to produce antibacterial textile [17]. Different from the Rajabi et al., work, we are going to utilize layered double hydroxide (LDH) as a substrate to produce homogeneous SONPs. In terms of structure, LDH is composed of a positively charged mixed metal hydroxide layer ((M(II)_1-x_M(III)_x_(OH)_2_)^x+^) and electrostatically stabilized interlayer anions [28,29]. Due to the various combination of metals and exchangeable interlayer anions, LDHs have been extensively studied as a platform for drug reservoirs [30,31,32] and a supporter in the hybrid catalyst [33,34]. Recently, Chen et al., reported that Ag_2_O could be formed on an LDH surface, utilizing LDH as a crystal growth substrate [35]. They mixed AgNO_3_ solution with a ZnAl-LDH suspension and NaOH was added to induce surface precipitation of Ag_2_O on the LDH surface. The LDH moiety in that report was utilized both as a substrate to grow Ag_2_O. In this contribution, we expanded the utility of LDH not only as a substrate to grow/immobilize Ag_2_O but also as a source of reactant and crystal-growth-modifier. For this purpose, the interlayer space of LDH was populated with citrate anions, which would be used as a capping agent to stabilize the crystal growth of Ag [36]. At the early stage of reaction, the edge of LDH is considered to be partially dissolved by the acidity of Ag^+^, resulting in the release of OH^–^ for Ag_2_O precipitation. Simultaneously, the citrate moiety would be provided from LDH. It is reported that the intercalated moiety in LDH structure is released in a controlled manner due to ion exchange property and periodic molecular arrangement in the gallery space of LDH [32,37,38]. Typically, the citrate supply would be mediated by the following general steps: (i) citrate at the periphery of the particle is released relatively fast, and (ii) the anions inside the particles would be released in a sustained manner. In this way, citrate moiety, which acted as a size controlling capping agent, would be slowly provided during the reaction between Ag^+^(aq) and OH^−^. The as-released citrate would readily cap the surface of SONP nuclei, preventing fast and random growth.

The current strategy could be realized as follows. Firstly, MgAl-LDH with interlayer citrate was synthesized as a substrate. Then, AgNO_3_ solution was reacted with the LDH suspension. The Ag/Al value, which is related to the Ag/LDH ratio, was varied to find the optimum condition for size control. The developed SONPs were characterized in terms of crystallinity, chemical purity, particle size, and electronic structure, to suggest citrate LDH as an appropriate substrate for SONP synthesis with controlled size. In addition, the antibacterial activity of the homogeneously developed SONP compared with heterogeneous bulk silver oxide was evaluated by disk diffusion and colony forming inhibition assay.

## 2. Materials and Methods

### 2.1. Materials

Magnesium nitrate hexahydrate (Mg(NO_3_)_2_·6H_2_O), aluminum nitrate nonahydrate (Al(NO_3_)_3_·9H_2_O), sodium citrate tribasic dihydrate (C_6_H_5_Na_3_O_7_·7H_2_O) and silver nitrate (AgNO_3_) were obtained from Sigma-Aldrich Co. LLC. (St. Louis, MO, USA). Sodium hydroxide (NaOH) was acquired from Daejung Chemicals and Metals Co. LTD. (Siheung, Gyeonggi-do, Korea). Luria–Bertani (LB) agar and LB broth were purchased from Becton, Dickinson and Company (BD, Sparks, MD, USA). All reagents were utilized without further purification.

### 2.2. Preparation of Pristine MgAl-Citrate LDH

Pristine LDH was prepared by the conventional co-precipitation method with citrate anion. Firstly, we mixed a solution of Mg(NO_3_)_2_·6H_2_O (0.16 M) and Al(NO_3_)_3_·9H_2_O (0.079 M) and added C_6_H_5_Na_3_O_7_·2H_2_O solution (0.47 M). Next, the mixed solution was titrated with 1.2 M NaOH solution under a nitrogen atmosphere until pH 9.5 was achieved. The suspension was stirred vigorously for 24 h at room temperature. After the reaction, the precipitate (MgAl-citrate LDH) was obtained by centrifugation, washing and lyophilization.

### 2.3. Preparation of Silver Oxide Nanoparticle Decorated LDH (SONP@LDH)

To immobilize SONPs on an LDH surface, an AgNO_3_ solution was reacted with LDH. For the reaction with different Ag/Al molar ratios of 1, 1/3, and 1/10, AgNO_3_ solution of the corresponding concentration 0.042, 0.014 and 0.0042 M was prepared. MgAl-citrate LDH powder (1 g) was thoroughly dispersed in 100 mL of each AgNO_3_ solution. Then the reaction was conducted under vigorous stirring at room temperature. After 24 h of the reaction, the precipitate was collected by centrifugation, washing and lyophilization. The SONP decorated LDH was represented as SONP@LDH-*n*, where *n* stands for the Ag/Al molar ratio in the reaction.

### 2.4. Characterization

In order to identify the structure of pristine MgAl-citrate LDH and SONP@LDHs, X-ray diffraction (XRD, Ultima IV, Rigaku, Tokyo, Japan) with a Cu Kα radiation (λ = 1.5406 Å) was utilized. Powder XRD patterns were measured in the 2 θ range from 5° to 80° with a scanning rate of 0.05°/s. Lattice parameters were determined using UnitCell software (Unitcell, Tim Holland and Simon Redfern, 2006, Cambridge University, Cambridge, UK) based on the acquired diffractograms. The chemical state of the silver was confirmed by X-ray photoelectron spectroscopy (XPS, K alpha+, Thermo VG, Waltham, MA, USA). The surface morphology of pristine MgAl-citrate LDH and SONP@LDH hybrids were observed using field emission-scanning electron microscopy (FE-SEM, Auriga, ZEISS, Oberkochen, Germany). The powder sample was dispersed in deionized water and drop-casted on a silicon wafer to prepare specimens for examination by scanning electron micrograph (SEM). Measurement was carried out at 15 kV acceleration voltage after Pt sputtering specimen for 60 s. In order to clearly identify the SONPs on SONP@LDHs, field emission transmission electron microscopy (FE-TEM) and selected area electron diffraction (SAED) pattern (Titan G2 ChemiSTEM Cs Probe, FEI, Hillsboro, OR, USA) were acquired. In conducting TEM, all samples were dispersed in deionized water with 10 min sonication and drop-casted on a Cu grid (200-square mesh gird with carbon film, Ted Pella, CA, USA). The TEM micrographs were acquired using a 200 kV acceleration voltage. To obtain the average particle size of SONPs, 50 particles were randomly selected from TEM images micrographs. The electronic structure of SONP@LDH hybrids was analyzed by UV-vis diffuse reflectance spectroscopy (EVOLUTION 220, ThermoFisher Scientific, Waltham, MA, USA) in the wavelength range from 200 to 1000 nm.

### 2.5. Antibacterial Activity Test

#### 2.5.1. Disk Diffusion Assay

The antibacterial activity of samples was first determined by a disk diffusion assay against a Gram-negative bacterium, *Escherichia coli* *(E. coli)* by following a previous report [39]. The biological resources used in this research were distributed from Korean Collection for Type Cultures (KCTC, Jeongeup, Korea). The *E. coli* was cultured in an LB agar plate in an incubator at 37 °C for overnight. Three colonies were inoculated in 5 mL of LB broth and cultivated in an incubator at 37 °C for 24 h. Commercially available bulk Ag_2_O with a heterogeneous particle size distribution (Silver (I) oxide, Sigma-Aldrich Co. LLC., St. Louis, MO, USA) was utilized as a reference sample.

Suspensions (10 mL) containing either bulk Ag_2_O or SONP@LDH (Ag_2_O concentration = 5 mg/mL) were sonicated for 30 min and 100 μL of each suspension was applied on a punched paper filter (6 mm, ADVANTEC, Toyo Roshi Kaisha Ltd., Tokyo, Japan). The sample-loaded paper filter was dried on a hotplate at 80 °C and stored in UV condition. Then, 20 mL of LB agar was poured into a petri dish and solidified at room temperature. *E. coli* bacterial suspension (LB broth medium, 10 μL of 1.7 × 10^5^ colony-forming units per mL) was uniformly spread on the surface of the LB agar plate. The LB agar plates were incubated at 37 °C for 24 h. The antibacterial activities were investigated by the diameter of the zone of inhibition around each paper disk. Each experiment was performed in triplicates and the standard deviations are presented as the final values.

#### 2.5.2. Colony Forming Inhibition Test

In order to quantify the antibacterial property of SONP@LDHs hybrids [40], colony-forming inhibition assay was carried out. Similar to Section 2.5.1, *E. coli* was selected and cultured. The bulk Ag_2_O and SONP@LDHs (Ag_2_O content was set the same at 10 mg) were added in 10 mL of *E. coli* bacterial suspension (1.7 × 10^5^ colony-forming units per mL). Each mixed suspension was incubated in an incubator at 37 °C for 24 h. Negative control samples were prepared without Ag moiety or with pristine MgAl-citrate LDH.

To estimate the number of bacterial cells in suspension, an optical density at 600 nm of the bacterial suspension (200 μL) was measured using a microplate reader (VARIOSKAN LUX, ThermoFisher SCIENTIFIC, MA, USA) [41]. In our work, 100-μL aliquots of the bacterial suspension were diluted 100 times and 20 μL was plated on an LB agar plate and incubated at 37 °C for 24 h. The colonies were counted on the next day. The experiments were performed in triplicates.

## 3. Results

### 3.1. Crystal Structure Analysis

**Figure 1:**
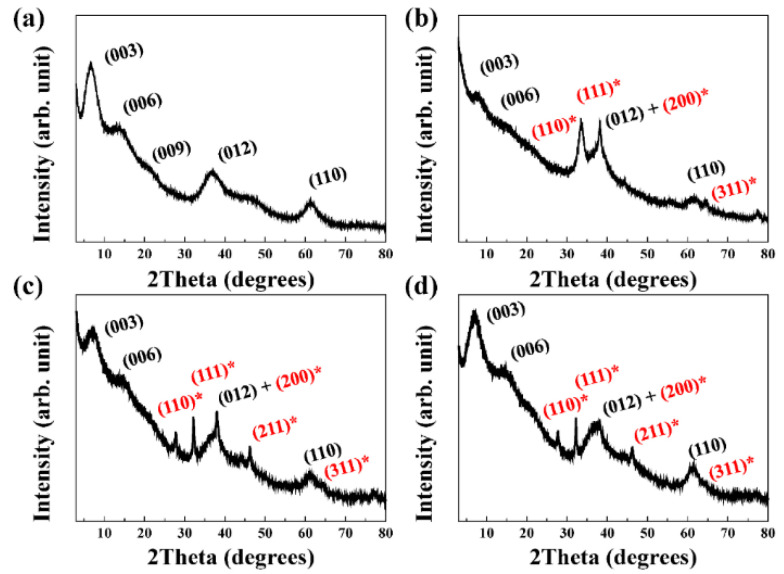
X-ray diffraction (XRD) patterns of (**a**) pristine MgAl–citrate layered double hydroxide (LDH), (**b**) Silver oxide nanoparticle (SONP)@LDH-1, (**c**) SONP@LDH-1/3 and (**d**) SONP@LDH-1/10 (black (hkl) notation: LDH, red(*) (hkl) notation: silver oxide).

The crystal structures of the pristine MgAl-citrate LDH and SONP@LDHs were confirmed using XRD patterns. The pristine LDH in Figure 1a exhibited well-developed (00l) peaks in low angle (2 θ < 25°) region and lattice peaks at high angle region (2 θ > 25°), which are in agreement with a hydrotalcite phase reported in the Joint Committee on Powder Diffraction Standards (JCPDS) card No.14-0191, indicating an LDH structure [42]. According to the corresponding (003), (006), and (009) peaks at 6.79°, 14.0°, and 21.4°, the d-spacing of the pristine MgAl–citrate LDH calculated by Bragg’s equation (*n*·λ = 2 d·sin θ, *n*: an integer (usually 1), d: d-spacing, θ: Bragg angle) [43] was 13.0 Å, which is slightly higher than a previously reported value of 12 Å [44]. This discrepancy was not serious as the d-spacing can be varied by degree of interlayer hydration. According to the molecular dynamics simulation report, the d-spacing of citrate-intercalated LDH could range between 9.39 and 15.6 Å, with a hydration number (*n*) between 3 and 12 (Mg_3_Al(OH)_8_(citrate)0.33·nH_2_O) [45]. As the literature suggested that hydration number and d-spacing had an almost linear relationship, we could estimate that the current MgAl–citrate LDH had hydration number 8–9. The degree of hydration is not discussed in detail here, as the MgAl–citrate LDH was subjected to aqueous reaction. Under water suspension, the hydration number does not have important meaning due to the dynamic changes in hydration state. Due to the sustained release property of LDH toward intercalated moiety [38,44], the citrate anion would be provided in a sustained manner during SONP formation. The acidity of AgNO_3_ partially dissolved LDH at the periphery, and the produced OH^−^ moieties, react with Ag^+^ (Equation (1) and (2)) [10]. The released citrate also reacted with Ag^+^ as a capping agent, also participating Ag_2_O crystal growth (Equation (3) and (4)) [36,46]. The combination of several chemical reactions is thought to inhibit the crystal growth of SONP.

Ag^+^(aq) + Mg_2_Al(OH)_6_^+^(s) ⇌ (1−x)Ag^+^(aq) + xAg(OH)(s) + Mg_2_Al(OH)_6−x_^(1+x)+^(s) (1)

2Ag(OH)(s) ⇌ Ag_2_O(s) + H_2_O(l) (2)

3 Ag^+^(aq) + (citrate)^3−^(aq) ⇌ Ag_3_(citrate)(aq) (3)

2Ag_3_(citrate)(aq) + 6OH^−^(aq) → 3Ag_2_O(s) + (citrate)^3−^(aq) + 3H_2_O(l) (4)

As expected above, the XRD patterns of SONP@LDHs in Figure 1b–d show characteristic patterns for Ag_2_O; peaks at 27.7°, 32.2°, 38.0°, 46.2°, and 64.5° attributed to (110), (111), (200), (211), and (311) of Ag_2_O (JCPDS card No.76-1393) [47,48]. These results suggested that a cubic crystal system with lattice parameter *a* = 4.81 Å was obtained for the synthesized SONP, although this is slightly larger than 4.76 Å for standard silver oxide (Ag_2_O), [49]. The slightly larger lattice parameter can be explained by the small particle size of oxide nanoparticles, which tended to show the expanded lattice compared with its bulk counterpart [50]. The presence of citrate as a capping agent rendered a negatively charged SONP, making it easy to electrostatically attach on a positive surface of LDH [51].

Although we could observe characteristic XRD peaks for LDH at the same position in all the SONP@LDHs, peak intensity and width were different from each other. The crystallinity of each sample was calculated by the Scherrer’s equation (t = 0.9 λ/Bcos θ, t: crystallite size (Å), λ: X-ray wavelength (Å), B; full-width at half-maximum of a peak, θ: Bragg angle) [52]. This has yielded a corresponding crystallite size of 2.63, 1.49, 2.13, and 2.50 nm along c-axis of LDH lattice for pristine LDH, SONP@LDH-1, SONP@LDH-1/3, and SONP@LDH-1/10. The results suggested that the larger quantity of Ag^+^ facilitated more dissolution of LDH, giving rise to a partial collapse of the LDH layer. Enhanced release of OH^−^ and citrate in SONP@LDH-1 influenced the formation of SONP, possibly resulting in unregulated crystal growth. As the XRD showed different crystallographic characters of LDH concerning for Ag/Al ratio, it is expected that the size and distribution of SONPs would be influenced accordingly, which will be discussed in the successive section.

### 3.2. Quantification of SONP@LDHs and Microscopic Analysis

Chemical compositions of samples were quantitatively evaluated with XPS and the results are summarized in Table 1. These results show that the amount of SONPs in the hybrid increased from 6.1% to 12%, and 23% for the corresponding Ag/Al ratio of 1/10, 1/3, and 1. The nominal Ag_2_O contents were estimated assuming all silver nitrate reacted to form Ag_2_O. The empirical Ag_2_O contents were determined by the quantification result of XPS measurement. However, these quantities of SONPs in the hybrid were less than the corresponding nominal value of 5.1%, 16% and 35%. The ratio between empirical to nominal Ag_2_O content was 0.66, 0.75, and 1.2, respectively, for Ag/Al ratio 1/1, 1/3, and 1/10. The tendency was due to the low availability of Ag^+^ when the Ag/Al ratio was high. Through the mismatch between nominal and empirical content, we could guess that the difference in Ag/Al ratio influenced how much Ag^+^ participated in the SONP formation. The difference would affect both surface property and particle morphology of SONPs. Therefore, we observed the morphology aspect in detail, utilizing electron microscopic techniques.

**Figure 2:**
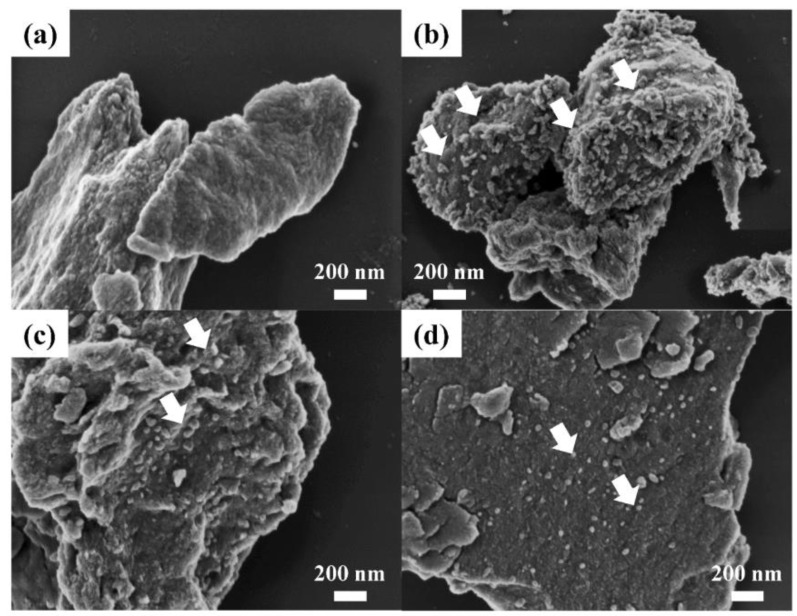
Field emission-scanning electron micrographs of (**a**) pristine MgAl–citrate LDH, (**b**) SONP@LDH-1, (**c**) SONP@LDH-1/3 and (**d**) SONP@LDH-1/10.

As shown in the scanning electron micrographs in Figure 2a, pristine LDH consisted of agglomerates of irregularly shaped small particles with a relatively smooth surface. On the other hand, as shown in Figure 2b–d, small particles assumed to be SONPs began to appear at the surface of large particles. The SONP@LDH-1 apparently exhibited the development of small particles on a large lump in Figure 2b. The number of particles decreased according to the diminishing Ag/Al ratio. In addition to the number of Ag_2_O particles, there is also a clear difference in morphology. In the scanning electron micrographs of SONP@LDH-1 in Figure 2b, particle size and shape of the SONPs were irregular with most of them forming aggregates of heterogeneous particles with sizes ranging from tens of nanometers to a few hundred nanometers (arrows in Figure 2). In Figure 2c, the number of irregular SONP particles decreased, but an aggregation of those particles was also observed. Particle size, shape, and distribution of small particles (SONPs) became homogeneous with decreasing Ag/Al ratio. The scanning electron micrographs in Figure 2d, exhibited that SONP@LDH-1/10 contained SONPs with homogeneous size and spherical shape.

**Figure 3:**
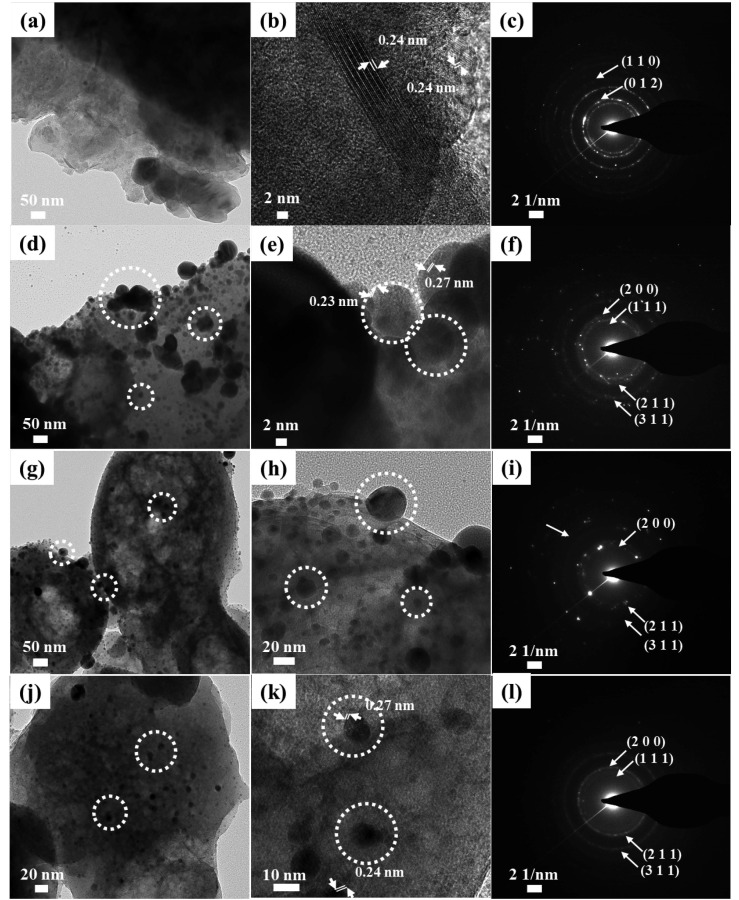
Transmission electron micrographs, higher magnification transmission electron micrograph (100,000×) with lattice fringes and selected area electron diffraction (SAED) patterns of pristine (**a**–**c**) MgAl–citrate LDH, (**d**–**f**) SONP@LDH-1, (**g**–**i**) SONP@LDH-1/3, and (**j**–**l**) SONP@LDH-1/10. Dotted circles stand for the SONPs.

To study in more detail the particle size of SONPs and their distribution, we carried out transmission electron microscopy. In Figure 3a, pristine LDH showed particles of tens of nanometers. We observed lattice fringe of which distance was 0.24 nm (Figure 3b). It indicated that the sample contained a crystalline array of certain planes with inter-plane distance 0.24 nm. From the XRD patterns of MgAl–citrate LDH (Figure 1a), we apparently observed (012) with d-spacing of 0.24 nm by Bragg’s equation. Therefore, we assigned that the observed lattice fringe was attributed to the arrangement of (012) planes in LDH. Furthermore, the selected area electron diffraction (SAED) pattern was obtained to show the existence of LDH. The SAED is a powerful tool to show the local crystal structure by diffracting the electron beam at a certain region. We observed clear ring patterns at 8.13 and 12.1 1/nm reciprocal lattice position (Figure 3c). The values in reciprocal space (SAED pattern) corresponded to the inter-plane distance of 0.123 and 0.0826 nm, respectively. These values well matched with the d-spacing values of (012) and (110) of LDH as observed in the XRD patterns (Figure 1a). Similar to the lattice fringe results above, the electron diffraction patterns in Figure 3c indicated the existence of crystalline LDH in the selected area. In Figure 3d, we observed particles with 39.45 ± 24.47 nm on a large platelet. In the higher magnification micrograph (100,000×) shown in Figure 3e, lattice fringes with distance 0.23 and 0.27 nm, respectively, were clearly observed. The inter-plane distance values matched with the d-spacing values of (200) and (111) in SONP. As the crystalline peaks, (200) and (111), were observed in XRD (Figure 1b), we could assign that the two lattice fringes were attributed to the arrays of (200) and (111) in SONP. The result confirmed that the particles with dotted circles in transmission electron micrographs were SONPs. The particle size and the standard deviation of SONPs became small with decreasing Ag/Al ratio: 20.75 ± 11.76 nm and 9.79 ± 3.23 nm for SONP@LDH-1/3 and SONP@LDH-1/10, respectively (Figure 3g,j). It was worth noting that the SONP@LDH-1/10 hybrid showed well-controlled Ag_2_O nanoparticles with narrow-sized distribution and homogeneous distribution. As described previously, we detected lattice fringes corresponding to SONPs in the TEM micrograph of SONP@LDH-1 (Figure 3e). The electron diffraction patterns of SONP@LDH samples (Figure 3f,I,l) at the particle region (dotted circles in Figure 3e,h,k) showed signals at 7.61, 8.37, 10.1, and 13.9 1/nm. The values in reciprocal space corresponded to 0.131, 0.119, 0.990, and 0.719 nm, respectively. These were the same with the d-spacing values of (111), (200), (211), and (311) in Ag_2_O. Therefore, we could conclude that the particles in transmission electron micrographs were SONP particles. To evaluate the distribution of SONPs, we gained energy dispersive spectroscopy mapping based on transmission electron micrograph (Supplementary Materials Figures S1–S4). As described above, SONP@LDH-1 and SONP@LDH-1/3 had a huge agglomerate of SONPs. On the other hand, SONP@LDH-1/10 showed a homogeneous distribution of Ag throughout the sample. Through these results, we could suggest that the Ag/Al ratio influenced the particle size of SONPs and controlled the distribution of particles.

### 3.3. Chemical Environments of Ag in SONP@LDHs

**Figure 4:**
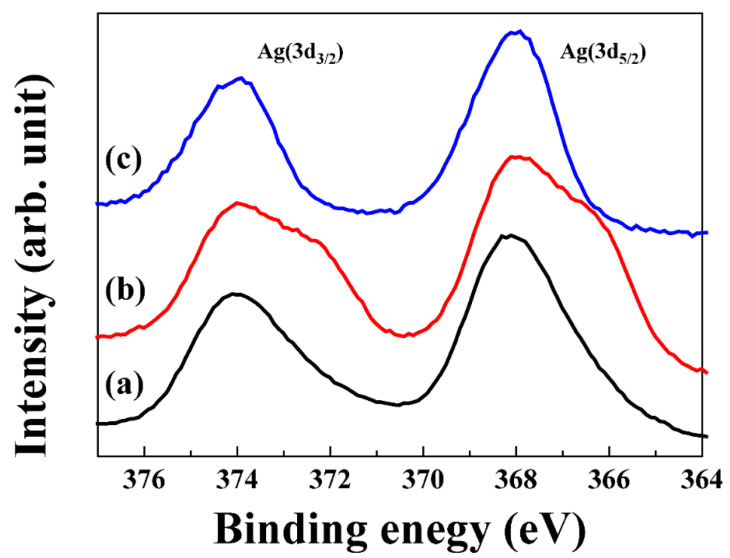
X-ray photoelectron spectroscopy (XPS) profiles for Ag 3d electrons of (**a**) SONP@LDH-1, (**b**) SONP@LDH-1/3 and (**c**) SONP@LDH-1/10.

The crystal structure of SONPs did not show a significant difference depending on Ag/Al ration in the XRD patterns and the SAED patterns. However, SEM and TEM observation presented that different particle morphology could be obtained according to Ag/Al ratio. The discrepancy was addressed by investigating the chemical environment of Ag utilizing XPS. As shown in Figure 4, the peak positions of all the samples were similar. The spectra for Ag 3d electrons exhibited two peaks at 374.03 (3d_3/2_) and 367.96 eV (3d_5/2_), which was well-matched with the binding energies of pure Ag_2_O at 373.90 (3d_3/2_) [53] and 367.7 eV (3d_5/2_) [54]. As the binding energy of an electron is strongly influenced by the chemical environment such as the type of adjacent atoms, and coordination number, the broad shape of the XPS peak suggested that the electrons are located in a complex chemical matrix. The shape of peaks differed depending on the Ag/Al ratio. The SONP@LDH-1 presented broad peaks, and SONP@LDH-1/3 exhibited humped peaks. The results implied that the SONP@LDH-1 and SONP@LDH-1/3 would have a complex chemical environment on Ag such as Ag(0) or AgO. Different formation processes of SONPs depending on the Ag/Al ratio might influence the chemical environment. At a low Ag/Al ratio, a large amount of LDH compared with Ag^+^ facilitated crystal growth of Ag_2_O in a controlled manner. Meanwhile, increasing the Ag/Al ratio would negatively affect the controlled crystal growth of Ag_2_O, resulting in disorganized phase and particle aggregation. The results suggested that the SONPs decoration on LDH surface with high purity and homogeneity depended on the ratio between LDH and Ag^+^ source.

### 3.4. Electronic Structure of SONP@LDHs

**Figure 5:**
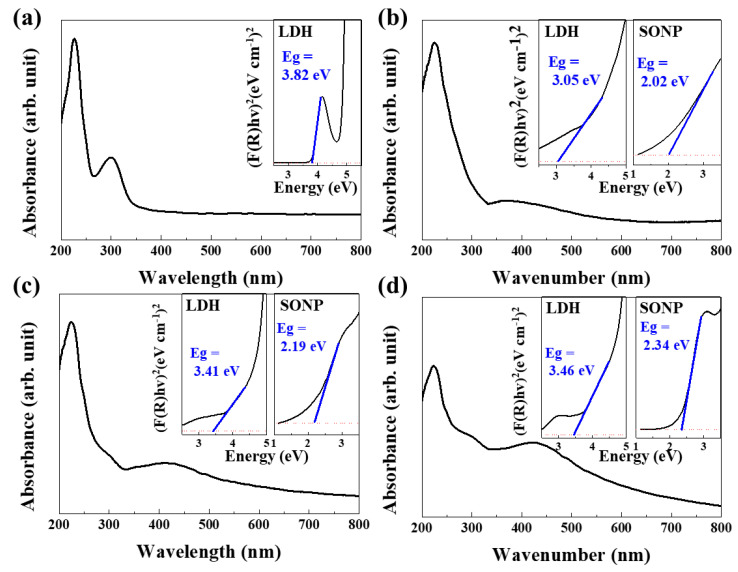
Diffuse reflectance UV-visible absorption spectra of (**a**) pristine MgAl–citrate LDH, (**b**) SONP@LDH-1, (**c**) SONP@LDH-1/3 and (**d**) SONP@LDH-1/10. Kubelka–Munk-transformed reflectance spectra and band-gaps were displayed as insets.

After pristine LDH reacted with AgNO_3_ solution, the color of the suspension changed gradually from white to pale grey, indicating the generation of Ag_2_O. To examine the electronic configuration of both LDH and SONP part in the hybrid, solid-state UV-vis spectroscopy was conducted. While pristine LDH showed two intense absorption peaks at 223 and 300 nm (Figure 5a), SONP@LDHs showed an intense peak at around 223 nm and broad absorption in the visible light region (Figure 5b–d). The broad absorption 300–600 nm has frequently been reported in the presence of Ag_2_O [55,56,57]. We expect to observe similar broad absorption (see Figure 5b–d) in the spectrum of SONP. Although all the SONP@LDHs exhibited similar light absorption patterns, we observed a difference in peak position depending on the Ag/Al ratio. To quantitatively analyze the difference, we converted the graphs to Kubelka–Munk reflectance spectra based on the Tauc formula (α*h*υ)^1/^^γ^ = B(*h*υ–E_g_), where α is the energy-dependent absorption coefficient, *h* is the Planck constant, υ is the photon’s frequency, E_g_ is the band-gap energy and B is a constant. The γ factor is the nature of the electron and is equal to 1/2 or 2 for direct and indirect transition band-gap, respectively [58]. The left and right panels of the inset in Figure 5b–d indicate the band-gap energy originated from LDH and SONP, respectively. The calculated band-gap values for SONPs in SONP@LDHs were correspondingly 2.02, 2.19, and 2.34 eV for Ag/Al ratio 1, 1/3, and 1/10, all of which are within the previously reported 1.3 to 2.35 eV range for Ag_2_O [1,2,3]. The band-gap energy is affected by both particle size and phase purity of Ag_2_O. It is generally known that the reduced size in nanoparticles increased band-gap energy [59,60,61]. Recent research on Ag_2_O reported that the existence of impurity or defect in Ag_2_O could decrease band-gap [62]. The smallest size (Figure 3j) and the highest purity (Figure 4c) of SONP at the Ag/Al ratio 1/10 rationalized the largest band-gap.

In addition to the band-gap change in SONP, we also observed that the band-gap energy of the LDH part was altered depending on the Ag/Al ratio. The M(OH)_6_ octahedron in LDH showed ligand-to-metal charge transfer excitations in the 200 to 300 nm range [63], the energy-gap being influenced by the crystallinity. Reportedly, LDHs with low crystallinity demonstrated a smaller band-gap than those with high crystallinity [64,65]. Parida et al. [65] reported decreasing band-gap energy from 2.80 to 1.86 eV depending on the crystallinity. In Figure 5a, the band-gap originated from LDH had 3.82 eV, while the corresponding hybrid samples correspondingly showed 3.05, 3.41, and 3.46 eV for SONP@LDH-1, 1/3, and 1/10, respectively. The band-gap of the LDH part reflected the crystallinity of LDH at different Ag/Al ratios.

### 3.5. Antibacterial Effect of SONP@LDHs

**Figure 6:**
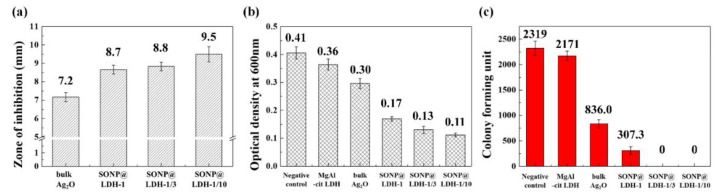
Antibacterial activity investigation by (**a**) zone of inhibition assay on LB agar plate, (**b**) optical density measurement at 600 nm after batch growth in liquid culture and (**c**) colony forming unit test on LB agar plate against *Escherichia coli* (*E. coli*.).

The advantage of homogeneous SONP particle size was clearly shown in the antibacterial test. For comparative study, commercially available bulk Ag_2_O with a heterogeneous particle size (Supplementary Materials Figure S5) was selected as reference material. First, a disk diffusion test was carried out to study the antibacterial activity of SONPs. Disks containing each sample were located on an agar plate where bacteria were inoculated. Upon incubation at an appropriate condition, the bacteria grew on the plate, except the periphery of antibacterial disks. The higher the antibacterial efficacy, the larger zone of inhibition around the disk is observed. As shown in Figure 6a and Supplementary Materials Figure S6, the SONPs prepared on LDH surface showed a larger zone of inhibition than bulk Ag_2_O. We also observed that the SONP@LDH-1/10, which showed the smallest and the most homogeneous SONP particles among the three samples, exhibited the largest inhibition zone for *E. coli*. As previously described [17], smaller SONP particles facilitated the release of Ag^+^ ions from the particle surface to the medium due to the large specific surface area. In this regard, SONP@LDH-1/10, which had the smallest SONP particles could effectively release Ag^+^ ions and accordingly showed a higher antibacterial effect on the contacted bacterial cells. The antibacterial mechanism of released Ag^+^ ions from SONPs has been proposed by several research groups. Due to the strong affinity of Ag^+^ ions for sulfur [66], the released Ag^+^ can interrupt disulfide bond or bind to thiol group in enzyme, resulting in the inactivation [67]. During the reaction between Ag^+^ and thiol or disulfide group, HS^−^ and S^2+^ are produced and they finally generate electrons through redox reaction. Through a set of reactions as described below, bacterial cells like *E. coli* consequently produce ROS such as O_2_^−^, OH^−^, OH and H_2_O_2_ that damage cell membrane proteins to suppress proliferation [68]. For example, Ag^+^ ions bound to succinate dehydrogenase or aconitase which contain iron-sulfur clusters, are known to promote the formation of hydroxyl radicals by the Fenton reaction [69]. Furthermore, the literature suggested that the thiol group occupied by Ag^+^ ions loses detoxification ability against ROS, finally resulting in bacterial suppression.

O_2_ + e → O_2_^−^ (5)

2O_2_^−^ + 2H^+^ → H_2_O_2_ + O_2_^−^ (6)

O_2_^−^ + H_2_O_2_ → OH^−^ + ·OH + O_2_^−^ (7)

Another antibacterial action of Ag^+^ is its interaction with nucleic acids [70]. Cellular internalized Ag^+^ can intervene in the triple or double hydrogen bonds of DNA base-pairs, giving rise to DNA damages or inhibition of DNA replication [68].

The antibacterial effect of SONPs was also demonstrated by directly adding the sample suspension before *E. coli* incubation. After incubation, the degree of antibacterial suppression was evaluated by measuring the number of cells or by counting the number of colonies. The number of bacterial cells can be estimated by measuring the optical density (absorbance) of bacterial solution at wavelength 600 nm. According to the previous literature [41], the optical density is directly proportional to the number of bacterial cells, where an optical density of 1.0 usually corresponds to approximately 1.0 × 10^8^ cells/mL. In the cell number evaluation, the optical density at 600 nm was measured after overnight incubation at 37 °C. Figure 6b exhibited that the *E. coli* without any treatment had fairly high optical density, which was directly proportional to the cell concentration. Both MgAl-citrate LDH and bulk Ag_2_O did not reduce the optical density of *E. coli* significantly. However, the three SONP@LDH prepared in this work showed a dramatic reduction in optical density. Among the three SONP@LDH, the one with the smallest SONP with the most homogeneous distribution (SONP@LDH-1/10) exhibited the lowest optical density. The antibacterial tendency was cross-confirmed by the colony-forming assays (Figure 6c and Supplementary Materials Figure S7). While bulk Ag_2_O showed an only ~2.7 times reduced number for the colony compared with negative control, SONP@LDH-1 exhibited a ~7.6 times lowered number of colonies. Furthermore, samples with more homogeneous SONPs such as SONP@LDH-1/3 and SONP@LDH-1/10, had 100% performance in colony inhibition. It should be noted here that MgAl–citrate LDH did not significantly affect colony-forming of *E. coli.* The result clearly showed that the SONPs prepared in this work increased antibacterial effect, due to the homogeneity in particle.

## 4. Conclusions

SONPs were successfully developed on the surface of LDH, while the intercalated citrate served as a size controlling moiety. The presence of Ag^+^ ions would partially cause the partial dissolution of the LDH lattice to release OH^−^ ions to form Ag_2_O. During the process, partially released citrate ions interacted with Ag^+^ to suppress the crystal growth of SONPs. According to the microscopic measurement, the Ag/Al ratio influenced the particle size and homogeneity of SONPs. In addition, XPS analysis revealed that the high Ag/Al ratio tends to produce SONPs with a complex chemical environment. UV-vis spectroscopy confirmed that the SONPs prepared at a low Ag/Al ratio was small in size and pure in composition. Moreover, SONP@LDHs with homogeneous particle size demonstrated significantly higher antibacterial activity against *E. coli* than bulk Ag_2_O with heterogeneous particle size. We suggest that the citrate-LDH could be utilized as a substrate to grow SONP, and that the Ag/Al ratio, i.e., Ag/LDH ratio, was a determining parameter to control particle size, distribution, and purity of SONPs. Furthermore, the antibacterial activity test clearly presented a close relationship with particle size and homogeneity.

## Figures and Tables

**Table 1:** Chemical formulae of pristine MgAl–citrate LDH, SONP@LDH-1, SONP@LDH-1/3 and SONP@LDH-1/10. Nominal composition was calculated by hypothesizing all Ag^+^ participated in SONP production. Empirical composition was the quantitative analysis results from XPS.

	Nominal Composition		Empirical Composition	
	Chemical Formulae	Ag_2_O Contents (%)	Chemical Formulae	Ag_2_O Contents (%)
MgAl–citrate LDH	Mg_1.6_Al(citrate)_0.33_(OH)_5.2_	-	Mg_1.6_Al(citrate)_0.33_(OH)_5.2_	-
SONP@LDH-1	Mg_1.6_Al(citrate)_0.33_(OH)_5.2_(Ag_2_O)_0.50_	35	Mg_1.5_Al(citrate)_0.33_(OH)_5.0_(Ag_2_O)_0.28_	23
SONP@LDH-1/3	Mg_1.6_Al(citrate)_0.33_(OH)_5.2_(Ag_2_O)_0.17_	16	Mg_1.5_Al(citrate)_0.33_(OH)_5.0_(Ag_2_O)_0.12_	12
SONP@LDH-1/10	Mg_1.6_Al(citrate)_0.33_(OH)_5.2_(Ag_2_O)_0.050_	5.1	Mg_1.4_Al(citrate)_0.33_(OH)_4.8_(Ag_2_O)_0.057_	6.1

## Data Availability

The data presented in this study are available on request from the corresponding author.

## Supplementary Materials

The following are available online at https://www.mdpi.com/2079-4991/11/2/455/s1, Figure S1: High-angle annular dark-field (HAADF) and TEM energy dispersive spectroscopic (EDS) mapping images of pristine MgAl–citrate LDH, Figure S2. High-angle annular dark-field (HAADF) and TEM energy dispersive spectroscopic (EDS) mapping images of SONP@LDH-1, Figure S3. High-angle annular dark-field (HAADF) and TEM energy dispersive spectroscopic (EDS) mapping images of SONP@LDH-1/3 and Figure S4. High-angle annular dark-field (HAADF) and TEM energy dispersive spectroscopic (EDS) mapping images of SONP@LDH-1/10. Figure S5. Field emission-scanning electron microscopy images of bulk Ag_2_O. Figure S6. Optical images of zone of inhibition assay for (**a**) negative control, (**b**) pristine MgAl–citrate LDH, (**c**) bulk Ag_2_O, (**d**) SONP@LDH-1, (**e**) SONP@LDH-1/3 and (**f**) SONP@LDH-1/10 containing paper on LB agar plates incubated at 37 °C 24 h. Each paper is 6 mm in diameter. Figure S7. Optical images of colony-forming unit test assay for (**a**) negative control, (**b**) pristine MgAl–citrate LDH, (**c**) bulk Ag_2_O, (**d**) SONP@LDH-1, (**e**) SONP@LDH-1/3 and (**f**) SONP@LDH-1/10 on LB agar plates incubated at 37 °C, 24 h.
