# Supplementary material for: Controlled Growth of Silver Oxide Nanoparticles on the Surface of Citrate Anion Intercalated Layered Double Hydroxide
Source: Nanomaterials (Basel). 2021 Feb 11;11(2):455. doi: 10.3390/nano11020455 (PMC7916874; doi:10.3390/nano11020455)
Supplement: Supplementary file 1 [file nanomaterials-11-00455-s001.pdf]

nanomaterials

Suplementary Materials: Controlled Growth of Silver Oxide Nanoparticles on the Surface of Citrate Anion Intercalated Layered Double Hydroxide

Do-Gak Jeung [1], Minseop Lee [2], Seung-Min Paek [2,*] and Jae-Min Oh [1,*]

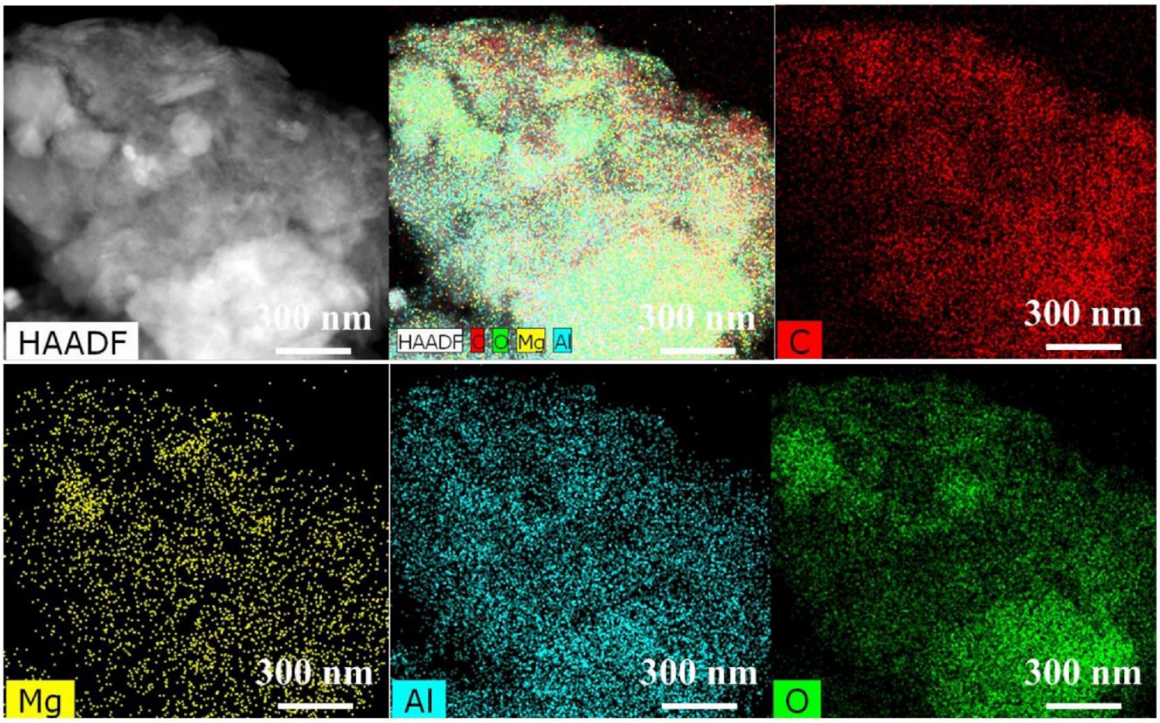

Figure S1. High-angle annular dark-field (HAADF) and TEM energy dispersive spectroscopic (EDS) mapping images of pristine MgAl-citrate LDH.

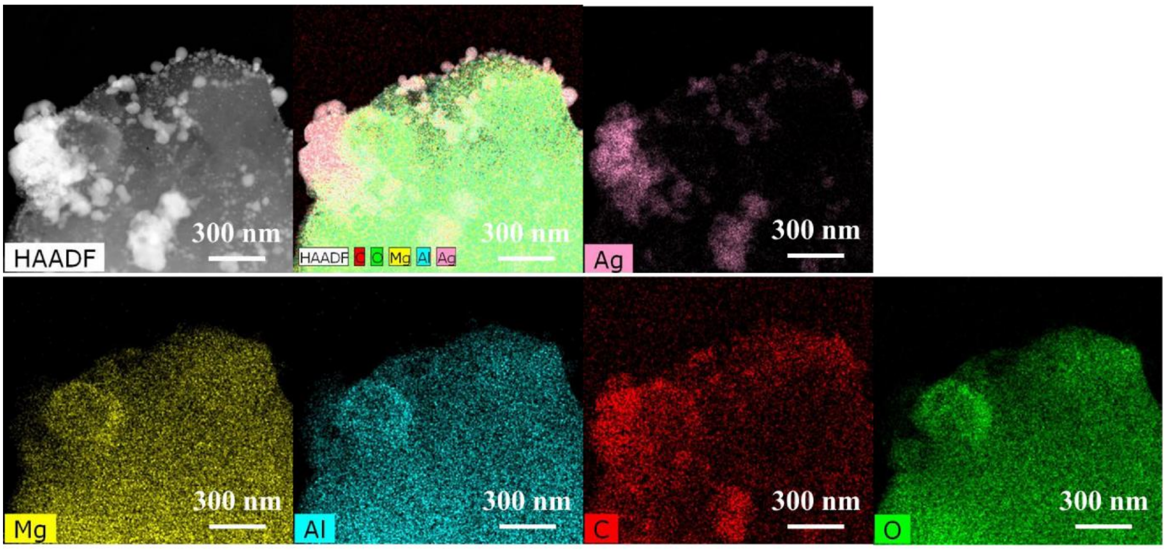

Figure S2. High-angle annular dark-field (HAADF) and TEM energy dispersive spectroscopic (EDS) mapping images of SONP@LDH-1.

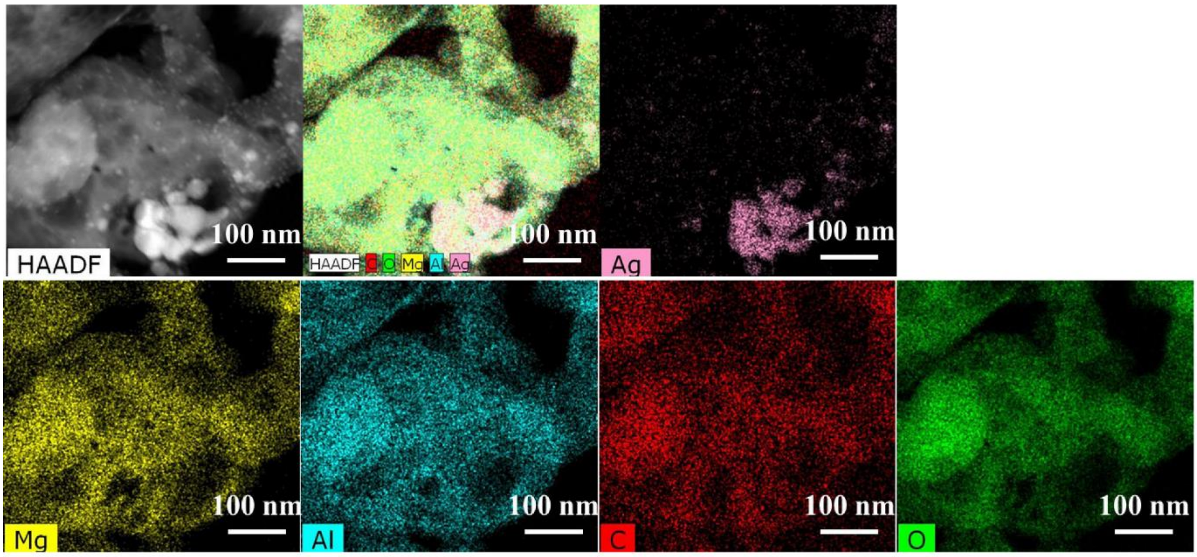

Figure S3. High-angle annular dark-field (HAADF) and TEM energy dispersive spectroscopic (EDS) mapping images of SONP@LDH-1/3.

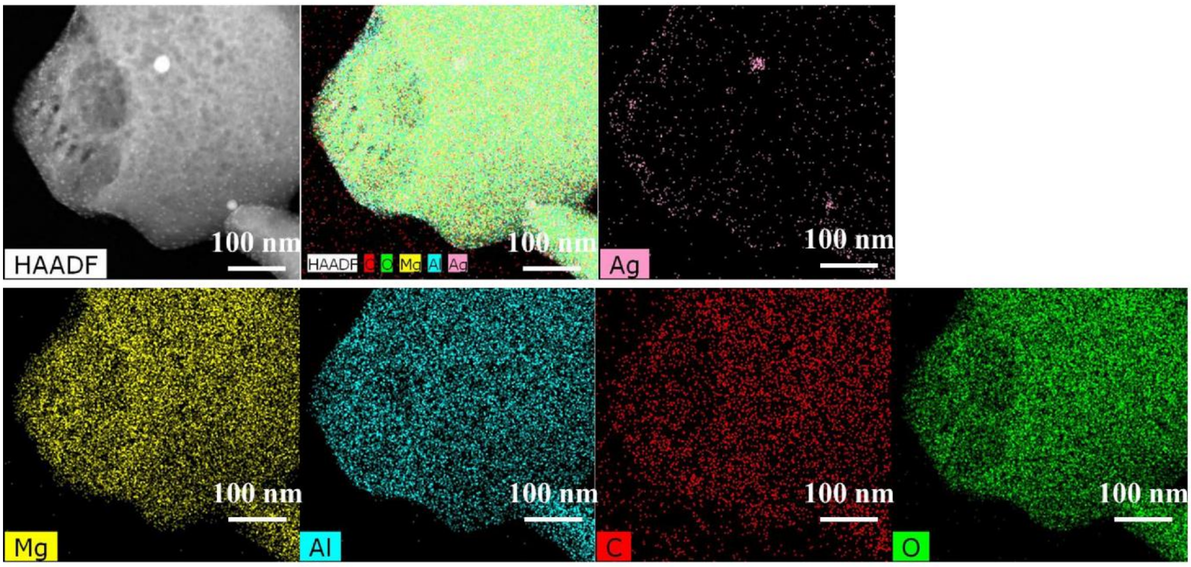

Figure S4. High-angle annular dark-field (HAADF) and TEM energy dispersive spectroscopic (EDS) mapping images of SONP@LDH-1/10.

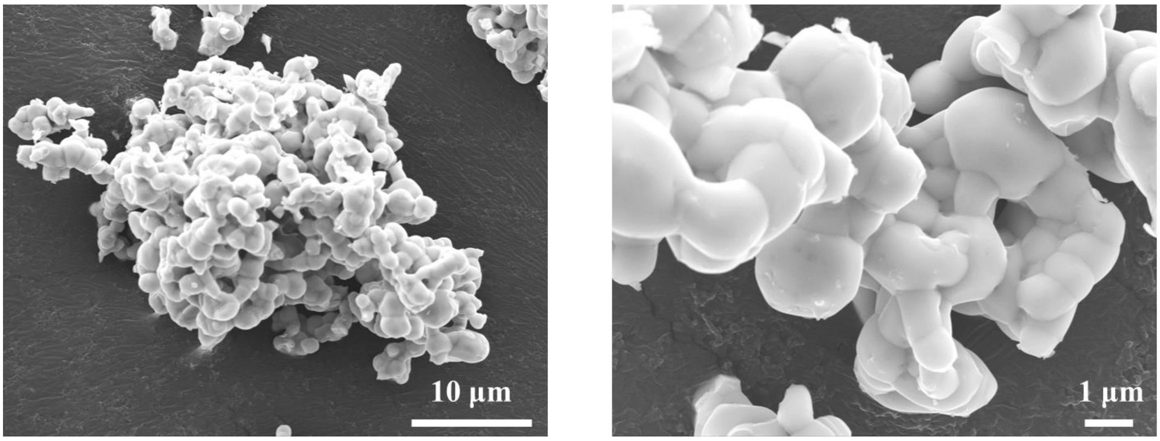

Figure S5. Field emission-scanning electron microscopy images of bulk Ag_2O.

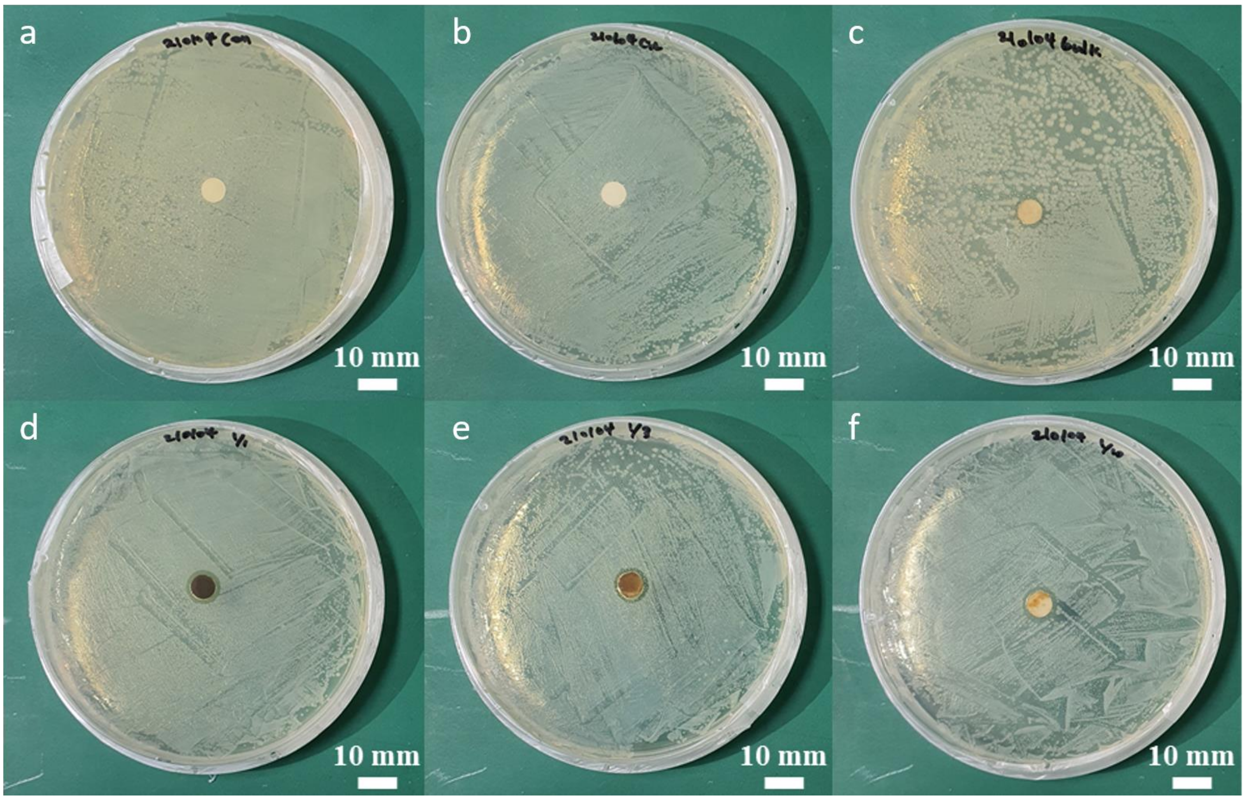

Figure S6. Optical images of zone of inhibition assay for (**a**) negative control, (**b**) pristine MgAl-citrate LDH, (**c**) bulk Ag_2O, (**d**) SONP@LDH-1, (**e**) SONP@LDH-1/3 and (**f**) SONP@LDH-1/10 containing paper on LB agar plates incubated at 37 °C, 24 h. Each paper is 6 mm in diameter.

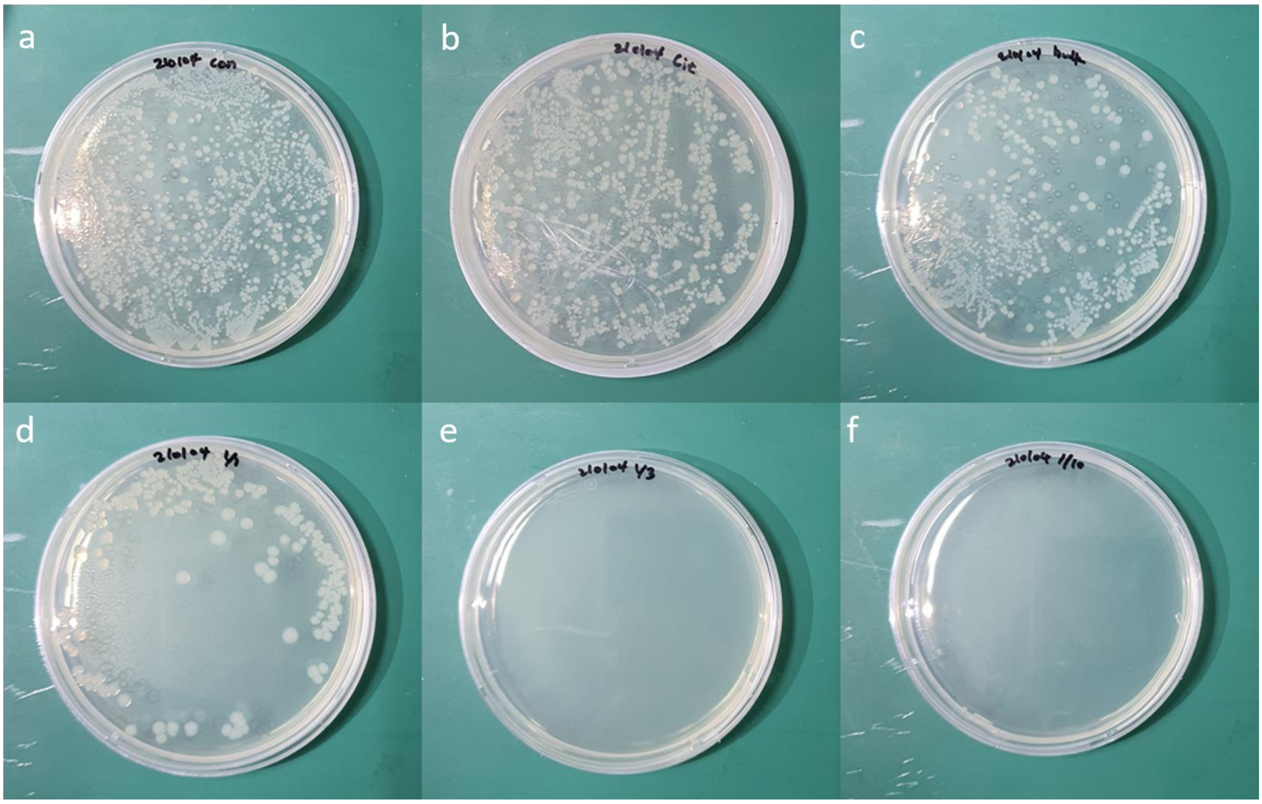

Figure S7. Optical images of colony forming unit test assay for (**a**) negative control, (**b**) pristine MgAl-citrate LDH, (**c**) bulk Ag_2O, (**d**) SONP@LDH-1, (**e**) SONP@LDH-1/3 and (**f**) SONP@LDH-1/10 on LB agar plates incubated at 37 °C, 24 h.
